# Physical Activity, Sedentary Behavior, and Diet-Related eHealth and mHealth Research: Bibliometric Analysis

**DOI:** 10.2196/jmir.8954

**Published:** 2018-04-18

**Authors:** Andre Matthias Müller, Carol A Maher, Corneel Vandelanotte, Melanie Hingle, Anouk Middelweerd, Michael L Lopez, Ann DeSmet, Camille E Short, Nicole Nathan, Melinda J Hutchesson, Louise Poppe, Catherine B Woods, Susan L Williams, Petra A Wark

**Affiliations:** ^1^ Domain: Health Systems & Behavioural Sciences Saw Swee Hock School of Public Healh National University of Singapore Singapore Singapore; ^2^ Sports Centre University of Malaya Kuala Lumpur Malaysia; ^3^ School of Health Sciences University of South Australia Adelaide Australia; ^4^ Physical Activity Research Group School of Health, Medical and Applied Sciences Central Queensland University Rockhampton Australia; ^5^ Department of Nutritional Sciences College of Agriculture and Life Sciences The University of Arizona Tucson, AZ United States; ^6^ EMGO Institute for Health and Care Research Department of Epidemiology and Biostatistics VU University Medical Centre Amsterdam Netherlands; ^7^ Texas A&M AgriLife Extension Service Texas A&M University College Station, TX United States; ^8^ Department of Movement and Sports Sciences Faculty of Medicine and Health Sciences Ghent University Ghent Belgium; ^9^ Research Foundation Flanders Brussels Belgium; ^10^ Freemasons Foundation Centre for Men’s Health Faculty of Health Sciences University of Adelaide Adelaide Australia; ^11^ Priority Research Centre for Health Behaviour School of Medicine and Public Health The University of Newcastle Australia Newcastle Australia; ^12^ Hunter New England Population Health Hunter New England Area Health Service Newcastle Australia; ^13^ Hunter Medical Research Institute Newcastle Australia; ^14^ Priority Research Centre in Physical Activity and Nutrition School of Health Sciences The University of Newcastle Australia Newcastle Australia; ^15^ Department of Physical Education and Sports Sciences Faculty of Education and Health Sciences University of Limerick Limerick Ireland; ^16^ Centre for Innovative Research Across the Life Course Faculty of Health and Life Sciences Coventry University Coventry United Kingdom

**Keywords:** science, telemedicine, exercise, health behavior, health resources, food, publications, movement, trends, Internet

## Abstract

**Background:**

Electronic health (eHealth) and mobile health (mHealth) approaches to address low physical activity levels, sedentary behavior, and unhealthy diets have received significant research attention. However, attempts to systematically map the entirety of the research field are lacking. This gap can be filled with a bibliometric study, where publication-specific data such as citations, journals, authors, and keywords are used to provide a systematic overview of a specific field. Such analyses will help researchers better position their work.

**Objective:**

The objective of this review was to use bibliometric data to provide an overview of the eHealth and mHealth research field related to physical activity, sedentary behavior, and diet.

**Methods:**

The Web of Science (WoS) Core Collection was searched to retrieve all existing and highly cited (as defined by WoS) physical activity, sedentary behavior, and diet related eHealth and mHealth research papers published in English between January 1, 2000 and December 31, 2016. Retrieved titles were screened for eligibility, using the abstract and full-text where needed. We described publication trends over time, which included journals, authors, and countries of eligible papers, as well as their keywords and subject categories. Citations of eligible papers were compared with those expected based on published data. Additionally, we described highly-cited papers of the field (ie, top ranked 1%).

**Results:**

The search identified 4805 hits, of which 1712 (including 42 highly-cited papers) were included in the analyses. Publication output increased on an average of 26% per year since 2000, with 49.00% (839/1712) of papers being published between 2014 and 2016. Overall and throughout the years, eHealth and mHealth papers related to physical activity, sedentary behavior, and diet received more citations than expected compared with papers in the same WoS subject categories. The Journal of Medical Internet Research published most papers in the field (9.58%, 164/1712). Most papers originated from high-income countries (96.90%, 1659/1717), in particular the United States (48.83%, 836/1712). Most papers were trials and studied physical activity. Beginning in 2013, research on Generation 2 technologies (eg, smartphones, wearables) sharply increased, while research on Generation 1 (eg, text messages) technologies increased at a reduced pace. Reviews accounted for 20 of the 42 highly-cited papers (n=19 systematic reviews). Social media, smartphone apps, and wearable activity trackers used to encourage physical activity, less sedentary behavior, and/or healthy eating were the focus of 14 highly-cited papers.

**Conclusions:**

This study highlighted the rapid growth of the eHealth and mHealth physical activity, sedentary behavior, and diet research field, emphasized the sizeable contribution of research from high-income countries, and pointed to the increased research interest in Generation 2 technologies. It is expected that the field will grow and diversify further and that reviews and research on most recent technologies will continue to strongly impact the field.

## Introduction

Being regularly active, having a less sedentary lifestyle, and consuming a healthy diet has many benefits for physical health, mental health, and well-being [1-3]. This is widely known, and the World Health Organization, the United Nations as well as many governments are committed to promoting these health behaviors [4]. Despite this, many people are not sufficiently active, are too sedentary, and/or do not adhere to dietary recommendations [5-7]. The negative consequences of the high prevalence of unhealthy behaviors are enormous for the individual, health care systems, and economies [1,6,8].

New technologies have been put forward as a cost-effective means to deliver behavioral health interventions and, as a result, prevent noncommunicable diseases (NCD) [9-12]. This is conceivable considering that the availability and personal use of information and communication technologies has increased significantly over the last two decades. Currently, 95% of the world population is covered by a mobile-cellular network and 84% is covered by a mobile-broadband network [13]. Although the Internet is still only accessible to 47% of the world population, access to the World Wide Web and smartphone usage across the globe is continuing to increase rapidly [13,14]. As such, there has been a rise in electronic health (eHealth) and mobile health (mHealth) related research for physical activity, sedentary behavior, and diet [10].

Thus far, eHealth and mHealth research related to physical activity, sedentary behavior, and diet has been summarized in several studies that focused on use and effectiveness of different technologies such as mobile phone and/or SMS (short message service) text messaging [15-18], digital games [19,20], the Internet [21-23], smartphone and/or tablet applications [24-28], social media [29], gamification features [30], and fitness trackers [31,32]. Other reviews in the field focused on specific populations such as children and adolescents [33,34], adults [35], older adults [36], overweight and obese adults [37,38], cancer survivors [39], patients with cardiovascular disease [40], and people residing in upper-middle, lower-middle, or low-income countries [41]. In addition, an international workshop addressed how eHealth and mHealth interventions should incorporate psychological theory and behavior change techniques in their design [42].

Although these studies summarized important aspects of the eHealth and mHealth research field related to physical activity, sedentary behavior, and diet, no attempt has been made to map out the entire field in a systematic manner. A bibliometric study uses publication-related information such as citations, journals, authors, and keywords to gain a bird's eye view of a field [43]. Bibliometric studies that summarized the research landscape in various fields have generated valuable insights [43-47] revealing the stage of maturity and growth of a research area, who and where the researchers are that drive the field, which journals are most prominent, and what kind of research is being conducted. This is especially useful in a relatively new research area such as eHealth and mHealth.

The purpose of this study is to examine the eHealth and mHealth research field related to physical activity, sedentary behavior and diet from its infancy until the end of 2016, and provide an overview of highly-cited papers which have considerably contributed to the maturation of the field. This will help researchers better position their work.

## Methods

### Search Strategy

We opted for using the Web of Science (WoS) Core Collection (Clarivate Analytics, USA) because it provides many bibliometric indicators and includes literature from most disciplines. We developed a search strategy in an iterative manner starting from search terms used in published reviews and literature already known to us. We refined the search strategy by screening the titles of the most accessed papers listed at websites of journals that publish in the research field, and the titles of all publications of 6 researchers from different countries that are highly active in the field (see Multimedia Appendix 1).

The final search was conducted on April 26, 2017 to ensure all relevant papers that were published between January 1, 2000 and December 31, 2016 were registered in the WoS Core Collection. We used 146 search terms related to (1) physical activity, sedentary behavior, and diet and (2) use of technology (eg, smartphone, Web). Terms were combined with Boolean Operators (“OR” within the two search domains, “AND” between the two search domains). We restricted the search to publications in English and did not search for book chapters, conference proceedings, book citation indexes, and chemical indices (see Multimedia Appendix 2 for the full search strategy).

A second search using the same terms was conducted in which we only retrieved papers that WoS marked as “highly cited.” WoS defines “highly cited” as being ranked within the top 1% compared with all other papers in terms of citation count in the same year and research field [48], suggesting highly-cited papers exert strong impact on the field.

The results of the two searches were exported to Microsoft Excel 2016 for screening.

### Screening of Search Results

We included all journal papers on eHealth and mHealth research related to physical activity, sedentary behavior, and/or diet (including proxies, eg, weight management). They comprisedeHealth and mHealth intervention studies; papers on the components or characteristics of eHealth and mHealth (eg, use of theory in apps); papers on the relationship between technology use and the health behaviors, validation studies of consumer-based assessment tools (eg, Fitbit); and papers on the development of eHealth and mHealth interventions targeting physical activity, sedentary behavior, and/or diet. Reviews, protocols, editorials, commentaries, and original research papers were eligible to gain a comprehensive picture of the field. We excluded papers that were not related to the field (eg, biology papers); reported that technology was only used for data collection (eg, Web-based surveys) or the delivery of education without trying to change behavior (eg, nutrition science course); or were related to validation of research-grade assessment technologies (eg, ActiGraph accelerometers). The detailed screening guide is presented in Multimedia Appendix 3.

We had earlier piloted the screening procedure. Coauthors screened the same set of 20 papers (selected at random from preliminary searches) using a protocol that described the inclusion and exclusion criteria and a tutorial video. The video introduced the overall concept of a bibliometric study compared with a systematic review and detailed the inclusion and exclusion criteria with examples to illustrate how they should be applied [49]. Coauthors indicated whether they would include or exclude a paper or were unsure, while consulting the video, abstract, and full-text upon demand.

Seven trained coauthors (AMM, CAM, CV, MH, MLL, ADS, and PAW) each received a unique set of papers for title screening with optional screening of the abstract and full-text. As in the pilot phase, they chose “include,” “exclude,” or the option “unsure.” Papers marked as unsure were screened by four of the authors (AMM, CAM, CV, and PAW) and discussed until consensus was reached.

### Bibliometric Analysis

We computed the (compound) growth rate of publications over time. This was done by raising the ratio of the number of publications in 2016 over those in 2000 to the power of 1/16, after which we subtracted one and multiplied by 100:

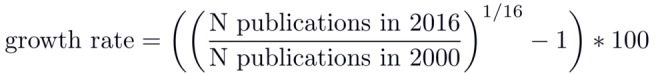



We calculated the citation rate by dividing the number of citations per publication by the time since publication until December 2016, and expressed this per year. The citation rate does not depend on time since publication and is therefore a more precise measure of a paper’s research impact than raw citation counts [50,51]. Because citation counts and citation rates are usually not normally distributed [47,52], we reported medians and interquartile ranges when studying their distributions.

Citation trends for physical activity, sedentary behavior, and diet related eHealth and mHealth research were studied between 2007 and 2016 because our analyses made use of published citation rates [53] that are only available over the most recent 10 years. We normalized the citation data for eligible papers by considering the WoS subject category and year in which a paper was published in two ways. First, we assessed the number of papers that occurred within each combination of WoS subject category and publication year among the papers included in our analysis. For each combination of WoS subject category and publication year separately, we multiplied the number of papers by the citation rate derived from the InCites Essential Science Indicator database on June 14, 2017 [53] to obtain the expected number of citations. After summing across WoS subject category, we obtained the total number of expected versus observed citations per year. Second, we compared the number of citations in each year and WoS subject category to corresponding published citation thresholds [53]. This yielded annual percentile scores that indicate the fraction of physical activity, sedentary behavior, and diet related eHealth and mHealth research articles within the top 10%, 20%, and 50% of all articles from the WoS subject categories represented by eligible papers in our search.

We explored the journals and authors who published most papers on eHealth and mHealth related to physical activity, sedentary behavior, and diet, along with the publication output of countries. We used WoS subject categories to count subject fields of papers. For the author analysis, we calculated 2 metrics using data within our dataset only: The *h*-index is the number of eligible papers of an author that were cited at least *h* times each (eg, an author with an *h*-index of 17 has at least 17 papers that were cited at least 17 times each) [54]. The *g*-index is the unique largest number of top cited eligible papers of an author that together received at least *g*^2^ citations (eg, the 17 top cited articles of an author with a *g*-index of 17 have at least 289 citations jointly) [55]. Countries were classified based on income as defined by the World Bank in 2017 [56].

To analyze the content of our dataset in more detail, we classified eligible papers into categories representing the studied exposure, technology, study population, setting, and methodology used. We did so by searching the title words and keywords identified by the author or by WoS editorial staff for occurrences of relevant terms. We defined the classification search terms using the agreed literature search strategy and the identified keywords in the eligible papers as starting point. The titles and keywords of the papers classified into each category were then double-checked by hand, as were those of all papers that were not classified into any or only a single category. This resulted in a refinement of the classification terms. This process was repeated until no inconsistencies were found. Using the final categorization reported in Multimedia Appendix 4, papers related to the Internet, (mobile) phone, SMS text messages, telehealth, and personal digital assistants were then classified as *Generation 1*, whereas papers on apps, wearable trackers, exergames, and social media were classified as *Generation 2*. Papers including both technologies were classified into *Generation 2*.

For the highly-cited papers, we also analyzed the papers based on their core content. These analyses were conducted independently by 2 coauthors (AMM and AM). They agreed on the descriptions of the paper’s core content by also using NVivo 11 (QRS International Pty Ltd, Doncaster, Australia).

We conducted descriptive analyses using Microsoft Excel version 2016 and the Bibliometrix package version 1.7 [57] for R version 3.3.3 (Vienna, Austria) [58]. We used Stata/SE version 14.2 (College Station, TX, USA) for keyword analysis.

## Results

### Results of the Search

**Figure 1:**
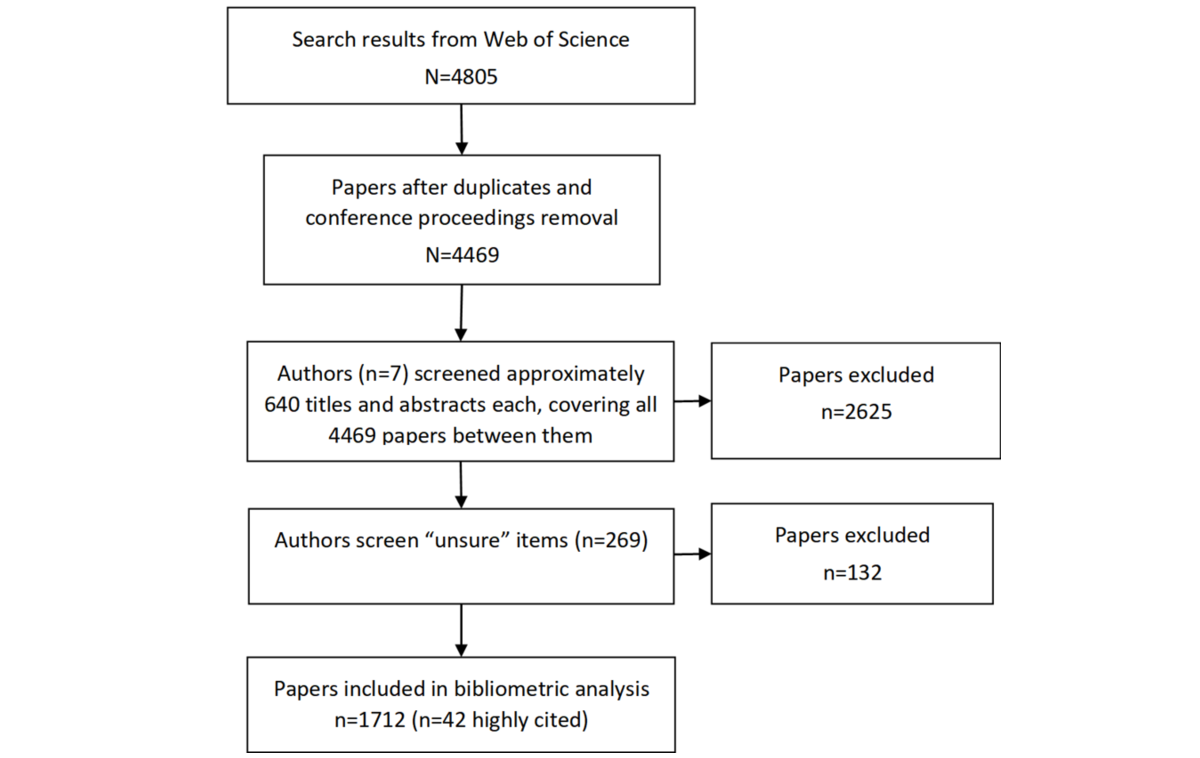
Screening flowchart.

Figure 1 displays the flow of the search and screening procedure. The search resulted in 4805 hits. Of these, 336 were duplicates or conference contributions. Their exclusion led to 4469 papers to be screened. A total of 1712 papers were included in the final bibliometric analysis (Multimedia Appendix 5), 42 of which were highly cited.

### Overall Trend

**Figure 2:**
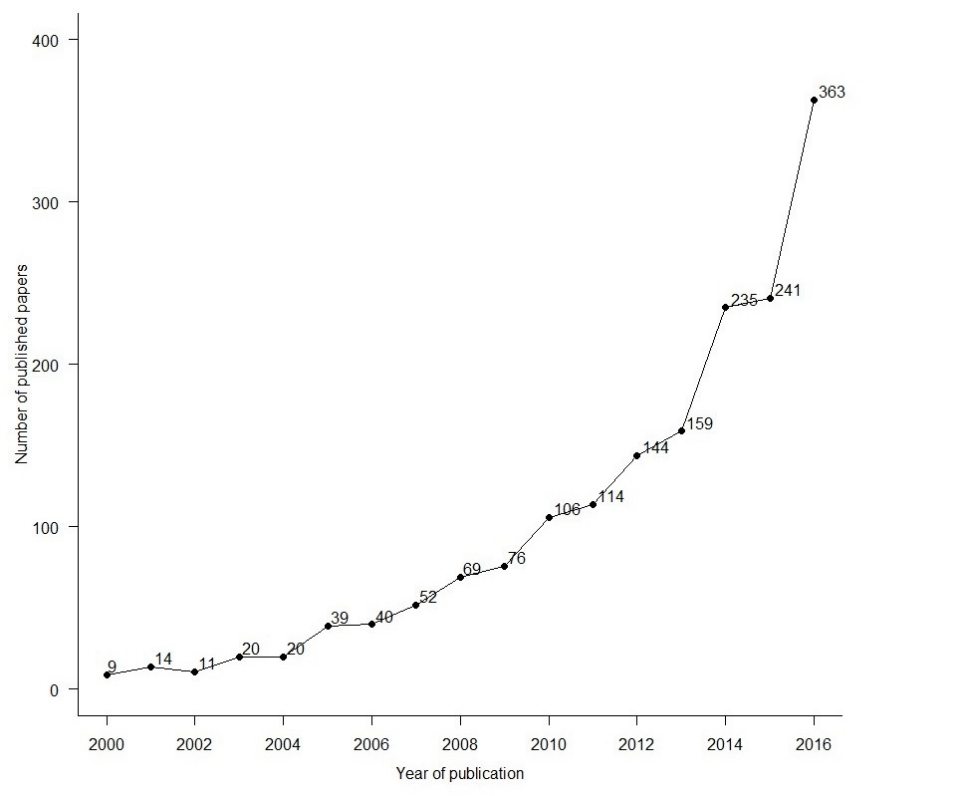
Publication output over time.

The number of papers on eHealth and mHealth related to physical activity, sedentary behavior, and diet increased steeply over the 17-year period (mean increase: 26% per annum). The period between 2014 and 2016 accounted for 49.00% (839/1712) of all papers (Figure 2).

The 1712 papers received 31,505 citations (median 7 per paper; interquartile range 18.5). Of the 1712 papers, 266 were not cited (15.54%), while 715 (41.67%) received 1 to 9 citations, 692 received 10 to 99 citations (40.42%), and 39 received 100 or more citations (2.28%). Overall, each paper received a median number of 2.0 citations per year (interquartile range 4.0).

**Figure 3:**
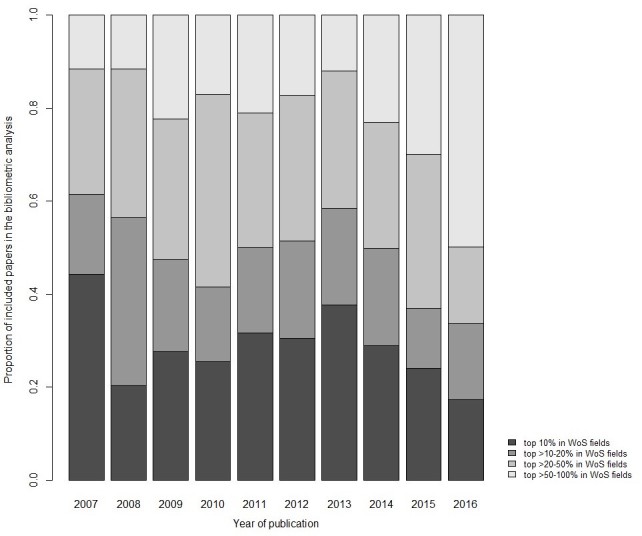
Distribution of physical activity, sedentary behavior, and diet related electronic health (ehealth) and mobile health (mHealth) research papers that were in the top 10%, 20%, 50%, and 100% cited among all papers from the same Web of Science (WoS) subject categories by year of publication.

Compared with all papers from the same WoS subject categories, the absolute number of citations was higher than expected for included papers (see Multimedia Appendix 6 for the WoS subject categories of included papers) in all studied years (ie, between 2007 and 2016; results not shown). However, the ratio of the expected to observed citations declined from 2.6 (95% CI: 2.5-2.7) in 2007 to 1.8 (95% CI: 1.6-2.0) in 2016. Half of the eHealth and mHealth papers related to physical activity, sedentary behavior, and diet remained in the top 50% cited papers across same WoS subject categories. However, the proportion of papers in the top 20% declined from 0.6 to 0.3 between 2007 and 2016 (Figure 3).

### Journals and Their Subject Categories

Overall, the papers were published by 471 different journals. As Table 1 shows, the *Journal of Medical Internet Research* published the most papers (9.58%, 164/1712 papers) followed by *BMC Public Health* (4.15%, 71/1712) and the *Games for Health Journal* (3.27%, 56/1712). The *Journal of Medical Internet Research* was also the highest cited journal and accounted for 13.48% of all citations in the field (n=4247 citations of 31,505 over the 17-year period). The *American Journal of Preventive Medicine* (9.37%, 2951/31,505) and *Annals of Behavioral Medicine* (4.64%, 1461/31,505) received the second and third highest number of citations, respectively.

In WoS, papers can be assigned to multiple subject categories. The papers included in this study were assigned to a total of 2797 WoS subject categories, of which 104 subject categories were unique. Table 2 shows the breakdown of subject categories present in the dataset, with only the top 10 categories shown. Throughout the years, the number of papers in journals from most fields gradually increased. However, the number of papers published in rehabilitation, health care science & services, health policy & services, as well as in medical informatics journals, increased more markedly from 2012. The number of papers published in psychology journals doubled between 2015 and 2016 (see Multimedia Appendix 6).

#### Authors

In total, 5654 authors contributed to the 1712 papers (median number of authors per paper 5, interquartile range 4). The top 10 authors (Table 3) contributed to 298 papers (17.41% of all papers). Vandelanotte C contributed to most papers (n=43) followed by Brug J (n=34), De Bourdeaudhuij I (n=31), and Oenema A (n=31). Vandelanotte C and Marcus B were in the top 10 of all characteristics listed in Table 3, and Brug J in all but first authorship papers.

#### Countries

As the country of the corresponding author usually indicates where the research originated, we could also analyze the origin of the research that was published in the field. Corresponding authors were from 46 countries (see Multimedia Appendix 7). Most papers were published by authors from the United States (n=836, 48.83% of all published papers) followed by authors from Australia (n=195, 11.39%) and the Netherlands (n=125, 7.30%). Overall, 96.90% (n=1659) of all papers published were authored by researchers from high-income countries. Of the remaining 53 papers, 45 were published by authors from 9 upper-middle income countries: China (n=21), Malaysia (n=7), Iran (n=4), Brazil (n=3), Turkey (n=3), Thailand (n=2), Lebanon (n=2), Romania (n=2), and Mexico (n=1). Papers published by authors from lower-middle income countries accounted for only 0.005% of all papers and came from India (n=4), Pakistan (n=1), Nigeria (n=1), Egypt (n=1), and the Philippines (n=1). Similar patterns appeared when considering coauthorship or first authors instead of corresponding authors (data not shown).

#### Keywords

Authors specified a total of 2448 different keywords across papers. After also adding the keywords specified by WoS editors, this resulted in 4283 unique keywords. The number of keywords per paper varied widely (median 12, interquartile range 6). A total of 43 papers lacked specification of any keywords. The keywords that were most used reflected the exposure (eg, “physical-activity”), the general topic (ie, “health”), the study design (eg, “randomized controlled-trial”), or the population (eg, “adults”). Several of the keywords, including commonly used ones, were uninformative by themselves (eg, “risk,” “program”). We were able to use the keywords in combination with title words to classify the content of the paper into categories.

**Table 1:** Journals publishing most papers in physical activity, sedentary behavior, and diet electronic- and mobile health (eHealth and mHealth) research (top 20).

Journals	Rank based on total output	Papers published (N=1712), n (%)	Rank based on total citations received from any journal	Citation count (N=31,505), n (%)	Impact factor 2016^a^	5-year impact factor^a^
*Journal of Medical Internet Research*	1	164 (9.58)	1	4247 (13.48)	5.175	5.835
*BMC Public Health*	2	71 (4.15)	7	640 (2.03)	2.265	2.814
*Games for Health Journal*	3	56 (3.27)	21	307 (0.97)	2.019	2.242
*JMIR mHealth uHealth*	4	50 (2.92)	26	240 (0.76)	4.636	4.463
*American Journal of Preventive Medicine*	5	40 (2.34)	2	2951 (9.37)	4.020	5.412
*International Journal of Behavioral Nutrition and Physical Activity*	6	38 (2.22)	8	529 (1.68)	4.396	5.813
*Journal of Nutrition Education and Behavior*	6	38 (2.22)	10	597 (1.89)	2.491	2.439
*Preventive Medicine*	8	29 (1.69)	4	1038 (3.29)	3.434	3.703
*Journal of Physical Activity and Health*	9	27 (1.58)	36	176 (0.56)	1.946	2.400
*Obesity*	10	25 (1.46)	5	1032 (3.28)	3.873	4.358
*Health Education Research*	10	25 (1.46)	6	781 (2.48)	1.816	2.183
*PLoS One*	12	24 (1.40)	28	238 (0.76)	2.806	3.394
*Telemedicine Journal and E-Health*	12	24 (1.40)	37	174 (0.55)	2.031	2.141
*Annals of Behavioral Medicine*	14	23 (1.34)	3	1461 (4.64)	2.976	4.508
*Computers in Human Behavior*	14	23 (1.34)	46	121 (0.38)	3.435	4.252
*Journal of Telemedicine and Telecare*	14	23 (1.34)	17	372 (1.18)	2.008	2.371
*JMIR Research Protocols*	14	23 (1.34)	68	79 (0.25)	N/A	N/A
*Patient Education Counseling*	18	21 (1.23)	16	374 (1.19)	2.429	3.042
*Translational Behavioral Medicine*	19	19 (1.11)	33	221 (0.70)	2.989	2.883
*American Journal of Health Promotion*	20	17 (0.99)	18	361 (1.15)	2.586	2.280

^a^Obtained from InCites Journal Citation Reports (Clarivate Analytics).

**Table 2:** Number of papers published in journals within the top 10 leading Web of Science (WoS) subject categories. Each paper can be assigned to multiple WoS subject categories (according to the categories specified at journal level).

WoS subject category	Different journals within WoS subject category, n	Papers in journals within WoS subject category (N=2797), n (%)
Public, environmental & occupational health	70	457 (16.34)
Health care sciences & services	32	346 (12.37)
Nutrition & dietetics	46	247 (8.83)
Psychology	53	217 (7.76)
Medical informatics	19	214 (7.65)
Medicine	30	145 (5.18)
Education & education research	8	103 (3.68)
Endocrinology & metabolism	30	96 (3.43)
Rehabilitation	28	94 (3.36)
Health policy & services	12	78 (2.79)

**Table 3:** Top 10 most published authors in electronic health (eHealth) and mobile health (mHealth) physical activity, sedentary behavior, and diet related research in either number of papers, first authored papers, citations, *h*-or *g*-index.

Author	All papers, n (rank)	First authored papers, n (rank)	Citations, n (rank)	*h*-index^a^ (rank)	*g*-index^a^ (rank)
Vandelanotte C	43 (1)	13 (1)	1379 (3)	17 (2.5)	37 (1)
Brug J	34 (2)	2 (180)	1666 (1)	19 (1)	34 (2)
Oenema A	31 (3.5)	3 (71)	980 (5)	12 (9)	31 (3)
De Bourdeaudhuij I	31 (3.5)	2 (180)	788 (9)	16 (4.5)	28 (5)
Marcus B	29 (5.5)	7 (4)	1089 (4)	16 (4.5)	29 (4)
De Vries H	29 (5.5)	1 (764.5)	404 (31)	11 (13)	19 (12.5)
Thompson D	27 (7)	10 (2)	696 (13)	11 (13)	26 (6)
Collins C	25 (8.5)	4 (30)	618 (17)	11 (13)	24 (7.5)
Maddison R	25 (8.5)	5 (15)	404 (31)	10 (18.5)	19 (12.5)
Morgan P	24 (10)	3 (71)	669 (15)	13 (7)	24 (7.5)
Tate D	23 (11)	3 (71)	1512 (2)	17 (2.5)	23 (9)
Eakin E	21 (13.5)	7 (4)	869 (7)	13 (7)	21 (10.5)
Baranowski T	21 (13.5)	6 (8)	748 (10)	11 (13)	21 (10.5)
Owen N	15 (22.5)	0 (1677.5)	955 (6)	13 (7)	15 (21.5)

^a^Within our dataset only.

**Figure 4:**
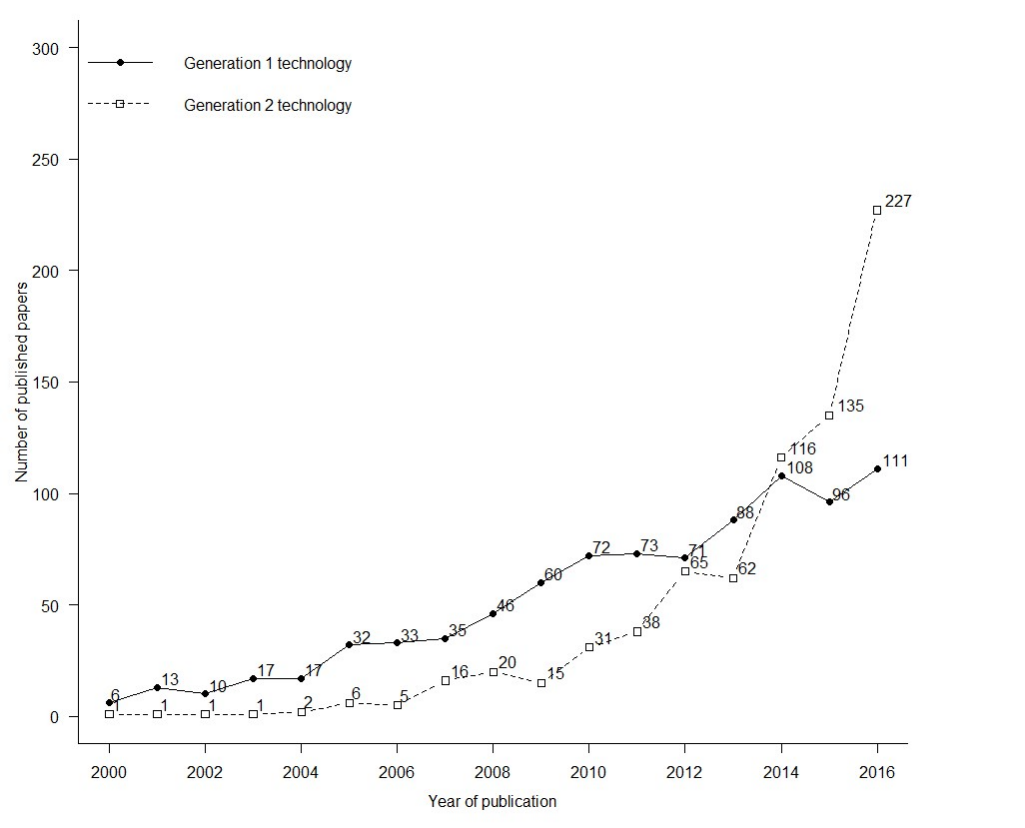
Number of published papers that studied Generation 1 technologies and Generation 2 technologies.

We classified 888 (51.87%) papers as studying *Generation 1* technologies and 742 (43.34%) papers as studying *Generation 2* technologies, with 82 (4.79%) papers being still unclassified. Before 2014, studies on *Generation 1* technologies were most common. From 2014 onwards, studies on *Generation 2* technologies were most common; their number steeply increased between 2013 and 2016. Within this period, the number of studies on *Generation 1* technologies increased less markedly (Figure 4). Vandelanotte C was the most common first author of papers on *Generation 1* technologies (n=11), followed by Harvey-Berino J (n=6). Gao Z was the most common first author of papers on *Generation 2* technologies (n=7), followed by Baranowski T (n=6).

Table 4 summaries the frequency of key study characteristics of the included papers. Physical activity was the health behavior most commonly targeted, followed by articles on weight and diet. Most studies targeted children or adolescents, while fewer focused on men and older adults. Multimedia and computer-based technologies (other than mobile apps) were most commonly studied, followed by studies focused on gamification or games, wearable technology or self-monitoring, or mobile apps or smartphones. Most studies were experimental trials, followed by reviews and/or meta-analyses. Few studies made use of creative or mixed methods.

#### Highly-Cited Papers

A table with all highly-cited papers can be found in Multimedia Appendix 8.

The 42 highly-cited papers received a total of 4883 citations (median 91, interquartile range 170) and were published between 2006 and 2016 across 19 journals. The *American Journal of Preventive Medicine* (n=13) and the *Journal of Medical Internet Research* (n=6) published most highly-cited papers. Corresponding authors were from nine countries, with US-based authors being the most common (n=20), followed by authors from Australia (n=8) and the United Kingdom (n=5). Overall, 226 authors contributed to the 42 highly-cited papers (mean 5.2 authors per paper) with Vandelanotte C (n=5) and Brug J (n=4) contributing to most highly-cited papers.

A systematic review and meta-analysis that reported on the link between intervention characteristics and intervention effectiveness on health behaviors (including physical activity and diet) in Internet interventions had the highest citation rate (79.5 citations per year) [59]. The authors included 85 studies and found that more extensive use of theory, a higher number of behavior change techniques and additional modes of communication (especially, text messages) were associated with larger effect sizes.

Of the 42 highly-cited papers, 20 were reviews of the literature, of which 19 used a systematic review approach, (4 of which also conducted a meta-analysis). A total of 13 papers reported data from primary studies and most of these were experimental trials such as randomized controlled trials (n=10); 8 studies reported content analyses of various smartphone apps, and 1 study introduced an eHealth and mHealth intervention development methodology. In terms of the technology used, 14 of the highly-cited papers studied *Generation 2* technologies, the majority of which related to social media, apps, or trackers (n=11). Most of the highly-cited papers were published in 2013 or later.

**Table 4:** Description of the physical activity, sedentary behavior, and diet related electronic health (eHealth) and mobile health (mHealth) research using expressions found in titles keywords of identified papers.

Category		Papers related to the category (as identified by its title or key words), n (%)^a^
**Modifiable factors**		
	Physical activity	1236 (72.19)
	Weight-related	859 (50.18)
	Diet or nutrition	621 (36.27)
	Sedentary behavior	169 (9.87)
**Technology**		
	Multimedia and computer-based technologies other than mobile apps	906 (52.92)
	Gamification or games	302 (17.64)
	Wearable technology or self-monitoring	251 (14.66)
	Mobile apps or smartphones	217 (12.68)
	Telehealth	191 (11.16)
	Text message	177 (10.34)
	Social media or marketing	89 (5.20)
**Populations**		
	Adults	555 (32.42)
	Adolescents or youth	406 (23.71)
	Children or infants	400 (23.36)
	Older adults	188 (10.98)
	Women	240 (14.02)
	Men	12 (0.70)
**Setting**		
	School or university	180 (10.51)
	Workplace	67 (3.92)
	Community	73 (4.26)
	Low- or middle-income countries, or low-income settings	48 (2.80)
	Family	37 (2.16)
**Research methodology/focus**		
	Experimental trial	813 (47.49)
	Review and/or meta-analysis	281 (16.41)
	Qualitative study	224 (13.08)
	Observational study	124 (7.24)
	Costs (including cost-effectiveness and financial incentives)	77 (4.50)
	Creative methods or designs	31 (1.81)
	Mixed methods (including Delphi studies)	10 (0.58)

^a^Terms are not mutually exclusive. We used text search to obtain the above categorization, which resulted in a hit if any part of the title or the author-defined and WoS-defined keywords had a specific phrase or word (see methods section and Multimedia Appendix 4). The presented values should thus be used as good indicators rather than absolute values.

## Discussion

### Principal Findings

The purpose of this paper was to examine the entirety of the eHealth and mHealth research field related to physical activity, sedentary behavior, and diet using bibliometric data. We observed a substantial growth of research output in the field with most papers being published in recent years.

An exponential growth pattern has been observed across all research disciplines. For example, there is a 2.3% increase in scientific publications per annum leading to a doubling of the publication volume every 24 years [60]. If the overall growth rate we observed also applies to the future, we can expect the publication volume in the physical activity, sedentary behavior, and diet related to eHealth and mHealth research field to double about every 4 years. This strong growth of research output may reflect the fast development, wide availability, and increased functionality and importance of modern technology in people’s daily lives. With this, opportunities to use these technologies to address behavioral health arise frequently. Across the world, researchers from various disciplines work on exploiting these new opportunities to understand and ultimately improve behavioral health. New intervention designs, methods, and analysis strategies are being developed [61-64]. We expect that these novel research initiatives will be widely disseminated in the scientific literature, which will likely lead to an increase in the research output. Compared with the entire body of research within the same WoS subject categories, eHealth and mHealth research related to physical activity, sedentary behavior, and diet was more frequently cited than expected. This likely signals an overall interest in the field.

The open access journals *Journal of Medical Internet Research* and *BMC Public Health* are the most popular outlets for researchers in this field. The *Journal of Medical Internet Research* was also the leading journal in an earlier bibliometric study that examined the overall mHealth literature [43]. The leading journals by citation count represent both open-access and nonopen access journals, with the *Journal of Medical Internet Research* receiving most and the *American Journal of Preventive Medicine* receiving the second most citations, respectively. Journals that publish open access enjoy a citation advantage in terms of speed of building up citations and overall citation count [65,66]. However, the size of this effect seems to be field-specific, which might explain why many nonopen access journals that publish physical activity, sedentary behavior, and diet eHealth and mHealth papers also accumulated a high number of citations [67,68]. The ranking of citation counts per journal is only a crude approximation of a journal’s impact, as journals that publish more papers enjoy more opportunities to receive citations.

While eHealth and mHealth research related to physical activity, sedentary behavior, and diet is maturing rapidly in many high-income countries, the research output of non-high-income countries is still meager. Only 3% of all papers were from upper-middle or lower-middle income countries. None of these countries published many papers in the English language journals included in WoS. It is possible that more papers are published in a local language or in journals not included in WoS. Nonetheless, this observation is unsatisfying, considering that (1) fast globalization and urbanization in many upper-middle and lower-middle income countries is related to reduced physical activity and unhealthy diets, which is associated with an unprecedented rise in NCDs seen in many of these countries [5,69,70] and (2) the (mobile) technology infrastructure is improving rapidly, implying that technology could be used effectively in settings with limited health care resources [71]. Although research in high-income countries is important, the largest public health impacts could be generated in upper-middle, lower-middle and low-income countries where about 80% of the world population lives. There are signs that physical activity and diet research is slowly increasing in some of these countries [72]. This is promising because resulting findings can be used to address these health behaviors at scale [41,71,73]. However, barriers related to funding, prioritization, research capacity and infrastructure, and language need to be overcome.

Although research related to relatively older *Generation 1* eHealth and mHealth technologies (eg, Internet, SMS text messages) accounted for most papers in the field, its annual growth rate was 20.0% compared with 40.4% for research using *Generation 2* technologies such as smartphone apps and wearables. This might indicate that the interest in *Generation 1* technologies in the research field is slowly declining as researchers and funding agencies prioritize *Generation 2* technologies. The trend of using the newest technologies to address health behaviors is expected to continue, but whether these technologies have a meaningful and long-lasting impact on people’s physical activity, sedentary behavior, and dietary habits needs to be seen [10]. Although, there are many arguments to be made for exploring very recent technologies, it is important to consider that these are currently often only available to a limited group of people. In addition, technologies may lose their appeal after a short time or are simply replaced by even newer technologies. These realities present barriers to achieving large-scale and sustainable public health impact with a specific technology.

We also found that most papers in the field were on physical activity compared with sedentary behavior and/or diet. That physical activity research is more common than research on dietary behaviors has been seen in the literature before [74,75]. Research on sedentary behavior is only a recent development as it was previously not distinguished from physical inactivity; a clear operationalization was only published recently [76]. Hence, to date, not many researchers have conducted eHealth and mHealth studies targeting sedentary behavior. Currently, only one review on eHealth and mHealth intervention studies targeting sedentary behavior exists [35].

We identified 42 highly-cited papers, which exert a strong influence on the field. These papers may be a resource for those less familiar with the field. The largest proportion of the highly-cited papers employed a systematic review approach to provide an overview of certain subfields. Systematic reviews and meta-analyses attract a high number of citations [77] as they are at the top of the evidence hierarchy in health-related subjects [78]. They are also important for researchers, policy makers, and practitioners alike. Intervention studies, such as randomized controlled trials, were also highly cited. However, their influence may decrease when more such studies in a specific area (eg, SMS text messaging interventions) accumulate and systematic reviews become available.

### Strengths and Limitations

The strength of this paper is that it provides a comprehensive overview of the eHealth and mHealth research field related to physical activity, sedentary behavior, and diet. To capture all relevant papers, we consulted the literature, discussed search terms, executed pilot searches, and refined our search before conducting the final search (with almost 150 search terms). We were similarly systematic in our paper-screening procedure. With this, we are confident that we have identified most eHealth and mHealth research papers related to physical activity, sedentary behavior, and diet.

We conducted a keyword analysis in combination with a title search to gain an insight into the studied exposures, technologies, populations, settings, and used methodologies. Because we did not confirm the obtained classification by retrieving the full-text, the number of papers for each category and subcategory we reported remains an estimate. However, this method allowed us to form an impression of the type of eHealth and mHealth research on physical activity, sedentary behavior, and diet that has been conducted between 2000 and 2016. Using keywords that are often used in a field is beneficial because others who consult databases to identify relevant research are likely to use these terms [79]. If authors use common keywords for their papers, database users are more likely to discover their work. This will also increase publication impact. Finally, in addition to providing an overview of the overall research in the field, we identified and analyzed highly-cited papers and highly published authors. This is important considering that, across all fields, only a small number of papers and authors determine the direction of a field [47,52].

Despite these strengths, limitations of our work need to be acknowledged. First, we only obtained papers from journals indexed by a single database—WoS. However, WoS is a large database that offers a wide variety of publication metrics that were vital for our analyses. Additionally, WoS only includes journals that meet certain criteria (eg, timely publishing, innovation, international diversity) to ensure high quality (more than 12,500 journals are currently indexed). Despite likely having excluded eligible papers in journals not included in WoS, we obtained papers from high-quality international journals that are the most influential source of scientific communication [52]. Using other databases such as Scopus could be explored in future studies. Second, we may have missed some papers that do not use informative keywords in the title as we did not search terminology used in abstracts. We did not review reference lists of eligible papers or their citations to identify any potentially missing papers. This, however, limited the (probably large) number of false positive results in our search. Third, we did not include gray literature (ie, conference proceedings, books, or other types of publications that are not journal papers), and we did not include papers published in languages other than English. Because of this, we may have missed relevant conference papers from fields such as human-computer interaction, computing, and engineering. Fourth, citation counts and their ranking should be interpreted with caution. Publication and citation habits vary between and even within fields [52]. The papers obtained for our bibliometric analysis were published in a variety of journals, and these journals are generally grouped under many subject categories (by WoS). Even though we considered the expected number of citations for each paper given its publication year and subject category when comparing citation trends over time, our approach remains an approximation: subject categories specified by WoS are assigned at journal level and may not reflect the field of every paper published in that journal. In addition, the citation counts we derived from WoS include self-citations, which may have influenced some of the rankings.

Finally, we have exclusively evaluated the scientific research literature and have identified trends that mainly concern scientific discovery, which will likely impact new research efforts. Therefore, the broader impact of the research outside of academia (eg, on public health, policy) cannot be deduced. Measuring and proving the societal impact of research is essential but difficult, mainly because this impact becomes usually only apparent in the far future and there are no agreed-upon measures to capture impact [80]. However, developments of measuring and analyzing impact beyond the scientific community are underway. One promising group of metrics that can be used are Altmetrics that measure the public engagement with research [81,82].

### Conclusions

In this paper, we provided a bird's eye view of the research on eHealth and mHealth related to physical activity, sedentary behavior, and diet. Our analysis of 1712 papers published between January 2000 and December 2016 showed that research output is increasing rapidly; a trend that appears likely to continue. The *Journal of Medical Internet Research* was highlighted as the primary outlet for research in the field. Despite the many promising developments, research in upper-middle, lower-middle, and low-income countries is still scant. More research in such settings is needed to examine the public health impact of eHealth and mHealth interventions on physical activity, sedentary behavior, and diet where needed the most. Systematic reviews and papers that report on recent technologies (mainly smartphone apps) exert a strong impact on the field and their influence will likely remain high in the future.

## Appendix

Multimedia Appendix 1 Journals and authors searched to refine our search strategy.

Multimedia Appendix 2 Search strategy (Web of Science).

Multimedia Appendix 3 Screening guide with inclusion and exclusion criteria.

Multimedia Appendix 4 Classification of search terms within titles and keywords identified by the author and WoS editorial staff into categories for content analysis.

Multimedia Appendix 5 File containing the included papers data from Web of Science.

Multimedia Appendix 6 Number of articles per subject category and publication year included in the bibliometric analysis.

Multimedia Appendix 7 Publication output of countries.

Multimedia Appendix 8 Highly-cited papers.

## Abbreviations

eHealth: electronic health
eHealth: electronic health
NCD: Noncommunicable disease
SMS: short message service
WoS: Web of Science
