# Supplementary material for: Physical Activity, Sedentary Behavior, and Diet-Related eHealth and mHealth Research: Bibliometric Analysis
Source: J Med Internet Res. 2018 Apr 18;20(4):e122. doi: 10.2196/jmir.8954 (PMC5932335; doi:10.2196/jmir.8954)
Supplement: Multimedia Appendix 1 [file jmir_v20i4e122_app1.pdf]

**Multimedia Appendix 1.** Journals and authors searched to refine our search strategy

**Journals**

- Journal of Medical Internet Research
- International Journal of Behavioural Nutrition and Physical Activity
- BMC Public Health
- American Journal of Preventive Medicine

**Authors**

- Hein de Vries
- Bess Marcus
- Anke Oenema
- Clare Collins
- Ralph Maddison
- Tom Baranowski
