# Supplementary material for: Physical Activity, Sedentary Behavior, and Diet-Related eHealth and mHealth Research: Bibliometric Analysis
Source: J Med Internet Res. 2018 Apr 18;20(4):e122. doi: 10.2196/jmir.8954 (PMC5932335; doi:10.2196/jmir.8954)
Supplement: Multimedia Appendix 2 [file jmir_v20i4e122_app2.pdf]

Multimedia Appendix 2. Search strategy

Advanced Search carried out on 26.04.2017

Limits: 2000-2016; Under ‘More Settings’: Core Collection (excluded book chapters, conference proceedings, book citation indexes, chemical indexes); languages: English; search in title

Search #1 (4609)
ti=(technolog* OR "video*" OR "CD-rom*" OR computer* OR podcast* OR www OR telephone* OR telemed* OR "tele med*" OR "tele-med*" OR telehealth OR "tele health" OR "tele-health" OR "communication technolog*" OR "information technolog*" OR "information-technolog*" OR ict OR "electronic health" OR "electronic-health" OR ehealth OR "e-health" OR internet* OR online* OR "on-line" OR "world wide web" OR "world-wide web" OR email* OR "e-mail*" OR website* OR "web-site*" OR "web-based" OR ipad* OR iOS OR "mobile health" OR mhealth OR "m-health" OR "digital" OR phone* OR "short message service" OR sms OR "text messag*" OR "multimedia messaging service" OR "picture messag*" OR mms OR "digital photograph*" OR app OR apps OR "mobile application*" OR multimedia OR PDA OR "personal digital assistant*" OR "handheld computer*" OR "hand-held computer*" OR "tablet PC" OR "tablet computer*" OR smartphone* OR "smart phone*" OR "smart-phone*" OR iphone* OR "mobile phone*" OR "mobile-phone*" OR "cell-phone*" or "cell phone" OR android OR tracker* OR wearable* OR "activity monitor*" OR fitbit* OR "apple watch" OR "mio fuse" OR "mio alpha" OR "mio link" OR "samsung gear" OR withings OR "misfit shine" OR "misfit flash" OR "jawbone up" OR "nike fuel band" OR "basis band" OR "garmin vivo" OR "social media*" OR blog* OR facebook* OR twitter OR tweet* OR youtube OR whatsapp OR Instagram OR "pokemon go" OR JITAI OR "just-in-time adaptive intervention*") AND ti=("physical activit*" OR "motor activit*" OR "outdoor activit*" OR exercis* OR walk* OR "active transport*" OR "active living" OR "leisure activit*" OR fitness OR acceleromet* OR pedomet* OR sedentar* OR "sitting time" OR "food intake" OR "sugar intake" OR "sugar consum*" OR "fat intake" OR "fat consum*" OR "sugar sweetened" OR "sugar-sweetened" OR "fizzy drink*" OR snack* OR "fruit* and vegetable*" OR "vegetable* and fruit*" OR TV OR television OR "tele-vision" OR "fruit* consumption" OR "fruit* intake" OR "vegetable* consumption" OR "vegetable* intake" OR "fast food*" OR "fast-food*" OR "take away*" OR "take-away*" OR "food habit*" OR "healthy eating" OR "dietary habit*" OR "eating habit*" OR "eating behavio*" OR diet* OR "diet* behavio*" OR nutrition* OR "carbonated beverage*" OR calor* OR "weight loss" OR "weight maintenance" OR "maintaining weight" OR "weight gain" OR "weight management" OR overweight OR obes* OR lifestyle* OR "behavio* change*" OR "behavio* intervent*")
Search #2 (196)
ti=(exergam*)
Search #1 OR #2 (4805), Highly cited: 61
