# Supplementary material for: Physical Activity, Sedentary Behavior, and Diet-Related eHealth and mHealth Research: Bibliometric Analysis
Source: J Med Internet Res. 2018 Apr 18;20(4):e122. doi: 10.2196/jmir.8954 (PMC5932335; doi:10.2196/jmir.8954)
Supplement: Multimedia Appendix 3 [file jmir_v20i4e122_app3.pdf]

Multimedia Appendix 3. Screening guide with inclusion and exclusion criteria

Please screen the titles in the spreadsheet and indicate if you would include or exclude them according to the definitions below. If you cannot make a decision based on the title go on to the abstract and decide. If you are still unsure after reading the abstract (or even full-text) then use blue to indicate that you are unsure; the core group will go through the titles that are marked ‘unsure’. Please use the following colour code.

Green=included

Blue=unsure

Red=excluded

Definitions

This is not a systematic review, hence, we are looking at a very broad picture: intervention studies, editorials, commentaries, reviews etc. related to e- & mHealth in physical activity, diet and/or sedentary behaviour (including proxies such as weight management).

Inclusion

1. Papers that describe the use of technology to impact PA, diet and/or sedentary behaviour; this also includes lifestyle interventions and papers in sport science (e.g., energy consumption of exergaming) and interventions targeting proxies (e.g. weight management, body mass index) as well as economic analysis of interventions, study protocols, usability, acceptability and feasibility studies (e.g., interest of parents to use technology for diet interventions; qualitative work around how participants perceived a physical activity app) and reviews/meta-analyses (including those on health behaviours).
2. Papers that describe components, aspects, use or characteristics of e- & mHealth technologies/research relevant for physical activity, diet and/or sedentary behaviour interventions (e.g., content analysis of diet smartphone apps; engagement with or use of physical activity trackers/diet apps).
3. Papers that describe the relationship between technology use and physical activity, diet and sedentary behaviour (including proxies such as weight

management; e.g., relationship between mobile phone use and sedentary behaviour); Must be aim of the research.

4. Validation studies of consumer assessment technologies (e.g., Fitbit, Garmin).
5. Papers that describe the development of a technology (e.g., website) that is intended to be used to impact physical activity, diet and/or sedentary behaviour.

Exclusion

1. Papers that are clearly not related to e- & mHealth for physical activity, diet and/or sedentary behaviour (e.g., papers on biology, chemistry or engineering).
2. Papers that describe the use of technology only for data collection and where there is no examination of how the data/technology relates to physical activity, diet and/or sedentary behaviour (e.g., online survey to examine dietary behaviour without any assessment how this online delivered survey impacted physical activity, diet and/or sedentary behaviour; online chat rooms about weight loss where there is no assessment on how this impacts physical activity, diet and/or sedentary behaviour).
3. Papers that describe the use of technology to deliver education without examining how this impacted physical activity, diet and/or sedentary behaviour (e.g., online course to nutrition science students).
4. Validation studies of research-grade physical activity and diet assessment technologies (e.g., assessing validity of research-grade accelerometers).
5. Book chapters, conference proceedings.
