# Supplementary material for: Physical Activity, Sedentary Behavior, and Diet-Related eHealth and mHealth Research: Bibliometric Analysis
Source: J Med Internet Res. 2018 Apr 18;20(4):e122. doi: 10.2196/jmir.8954 (PMC5932335; doi:10.2196/jmir.8954)
Supplement: Multimedia Appendix 4 [file jmir_v20i4e122_app4.pdf]

Multimedia Appendix 4. Classification of search terms within titles and keywords identified by the author and WoS editorial staff into categories for content analysis

Category	Subcategory	Phrases searched in the title or keywords[a]
Exposure (Modifiable factors)	Physical activity	“physical activ” OR “physical-activ” OR “physical edu” OR “physical-edu” OR (“activ” NOT (“enactive” OR “activities” OR “trans” OR “iveness” OR “inter-activ” OR “interactiv” OR “pro-activ” OR “proactiv” OR “re-activ” OR “reactiv” OR “brain” OR “activat” OR “active cop”) OR “exercis” OR “sport” OR “walk” OR “fitness” OR “front-crawl” OR “front crawl” OR “biking” OR “skate” OR “pedomet” OR “step” OR “acceler” OR “actigraph” OR “actograph” OR “body movement” OR “exergam” OR "pokemon go” OR “yoga” OR “run test” OR “wii” OR “aerobic dance” OR “dance”[b] OR “dancing” OR “aerobic fitness” OR “aerobic power” OR “aerobic training” OR “swim” OR “calorie expenditure” OR “ergomet” OR “energy-expenditure” OR “energy expenditure” OR “fitspiration” OR “fit”[b] OR “gait” OR “ipaq” OR “leisure-time pa” OR “tracker” OR “wearable” OR “activity monitor“ OR “activity track” OR “fitbit” OR “fit bit” OR “fit-bit” OR “apple watch” OR ("mio” AND “fuse") or ("mio” AND “alpha") or ("mio” AND “link") OR “samsung gear” OR “samsung-gear” OR “withings” OR “misfit shine” OR “misfit-shine” OR “misfit flash” OR “misfit-flash” OR “jawbone” OR ("nike” AND “fuelband“) OR “basis band “ OR “basis-band “ OR “basisband“ OR ("garmin” AND “vivo") OR “sensewear” OR “smart watch” OR “smartwatch” OR “smart-watch” OR “pedomet” OR “pedo-met” OR “yuuhokei” OR ("step” AND “watch") or ("step” AND “count") OR “activpal” OR “actiheart” OR (“ambulation” NOT “disorder”) OR “par-q” OR “time pa” OR “personal trainer” OR “mvpa” OR “telemove” OR “lifecorder” OR “locomotion” OR “treadmill” OR “movement

Category	Subcategory	Phrases searched in the title or keywords[a]
		skill" OR "human movement" OR "resistance train" OR "ambulatory status" OR "physical perform"
	Weight-related	"weight" OR "overweight" OR "obese" OR "obeso" OR "obesi" OR "adipos" OR "bmi" OR "waist circumference" OR "waist-circumference" OR "body composition" OR "body fat" OR "body-fat" OR "body mass" OR "body-composition" OR "body-mass" OR "energy balanc" OR "fat loss" OR "visceral fat" OR "nhf-nrg"
	Diet or nutrition	"food" OR "sugar" OR "fat intake" OR "fat consum" OR "low-fat" OR "saturated fat" OR "saturated-fat" OR "sugar" OR "vegetab" OR "fruit" OR "fast food" OR "take away" OR "take-away" OR "healthy eating" OR "eat healthier" OR "snack" OR "5 a day" OR "diet" OR "eating" OR "nutrit" OR "carbonated" OR "beverage" OR ("calor" NOT "direct") OR "intake" OR ("consum" NOT ("consumer-based" OR "consumer based" OR "consumer health" OR "consumer-health" OR "oxygen")) OR "energy dens" OR "energy-dens" OR "carboh" OR "caroten" OR "zinc" OR "potassium" OR ("protein" NOT (hemoglobin a1c protein" OR "lipoprotein" OR "reactive")) OR "fibre" OR "fiber" OR "dairy" OR "dash" OR "olive" OR "coffee" OR "tea"[c] OR "olive" OR "breakfast" OR "meal" OR "lunch" OR "dinner" OR "dish" OR "appetite" OR "satiety" OR "satiation" OR "fullness" OR "hunger" OR "hungry" OR "palatab" OR "restaurant" OR "supermarket" OR "farmers market" OR ("supplement" NOT "supplemented") OR "vitamin" OR "weighed record" OR "ketogenic" OR "myplate" OR "portion size" OR "efnep" OR "tfeq" OR "ffq" OR "glycemic index" OR "glycemic load" OR "goldberg cutoff"
	Sedentary behavior	"seden" OR "sitting" OR "stationar" OR "screen time" OR "screen-time" OR "television" OR "tele-vision" OR "tv" OR "computer time" OR "viewing time" OR "viewing behav"

Category	Subcategory	Phrases searched in the title or keywords[a]
Technology	Multimedia and computer-based technologies other than mobile apps	"web" OR "internet" OR "online" OR "on-line" OR "www" OR ("computer" NOT "wearable") OR "video" OR "multimedia" OR "multi-media" OR "cd-rom" OR "cdrom" OR "cd rom"
	Gamification or games	"game" OR "gaming" OR "gamif" OR ("augment" AND "realit") or "virtual" OR "nintendo" OR "kinect" OR "wii" OR "avatar" OR "exergam" OR "advergam" OR "pokemon go" OR "animat" OR "interactive dance"
	Mobile apps or smartphones	"apps" OR ("app" AND "mobile") or ("app" AND "cell") OR ("app" AND "smart") OR ("app" AND "phone") OR ("app" AND "ipad") OR ("app" AND "iphone") or ("app" AND "tablet") OR ("app" AND "handheld") OR ("app" AND "activity") OR "app"[b] OR app-based intervention" or "mobile fitness apps" OR "iphone" OR ("phone" AND "smart") or "android" OR "ios" OR "ipad" OR "tablet pc" OR "tablet computer" OR ("mobile" AND "dev")
	Wearable technology or self-monitoring	"tracker" OR "tracking" OR "tracks" OR "tracked" OR "wearable" OR "activity monitor" OR "activity track" OR "fitbit" OR "fit bit" OR "fit-bit" OR "apple watch" OR ("mio" AND "fuse") OR ("mio" AND "alpha") OR ("mio" AND "link") OR "samsung gear" OR "samsung-gear" OR "withings" OR "misfit shine" OR "misfit-shine" OR "misfit flash" OR "misfit-flash" OR "jawbone" OR ("nike" AND "fuelband") OR "basis band" OR "basis-band" OR "basisband" OR ("garmin" AND "vivo") OR "smart watch" OR "smartwatch" OR "smart-watch" OR "acceler" OR "pedomet" OR "yuuhokei" OR "pedo-met" OR ("step" AND "watch") OR ("step" AND "count") OR "activpal" OR "actiheart" OR "actograph" OR "actigraph" OR "self monitor " OR "self-monitor" OR "selfmonitor" OR "self-weigh" OR "selfweigh" OR "self weigh" OR "body-worn sensor" OR ("sensor" NOT ("sensory" OR "sensori")) OR "sensing" OR

Category	Subcategory	Phrases searched in the title or keywords[a]
		“sensecam” OR “camera" OR “actiheart” OR “lifecorder” OR “quantified self” OR “quantified-self”
	Telehealth	("tele" AND "health") OR (“tele” AND “med”) OR (“tele” AND “cardiol”) OR(“tele” AND “care”) OR (“tele” AND “dermatol”) OR (“tele” AND “nurs”) OR (“tele” AND “nutrition”) or (“tele” AND “psychiat”) OR (“tele” AND “support”) OR “home health” OR “skype” OR “telemove” OR “conference call” OR “automated call” OR (“remot” AND (“care” OR “deliver”))
	Text message	"messag” OR "message service” OR "text messag” OR "messaging service” OR "sms” OR "texting” OR "text-mess” OR ("text” NOT “context”) OR "mms” OR “email” OR "e-mail" OR “electronic mail” OR “electronic communication”
	Social media or marketing	("social med” NOT (“psycho-social” OR “psychosocial”)) OR "blog” OR "facebook” OR "twitter” OR "tweet” OR "youtube” OR "whatsapp” OR "instagram” OR "new media” OR "podcast" OR “networking site” OR “forum” OR “chat” OR “social market” OR “social netw”
	Phone (but not smart-phone)[d]	“phone” NOT “iphone” NOT (“smart”) NOT (“ipad”) NOT “android”
	Personal Digital Assistant[d]	“pda”[b] OR “personal digital assistant" OR “handheld computer” OR “hand-held computer”
Study Population	Adults	(“adult” NOT (“older-adult” OR “older-adult”)) OR student” OR “freshman” OR “"college-age” OR "college age" OR “employee” OR “worker” OR ("middle-aged men" OR "gentleman” OR "gentlemen” OR “scottish man” OR “mid-life” OR “midlife”
	Adolescents or youth	"adolesc” OR “youth” OR "young” OR "teen” OR “classmate”
	Children or infants	"baby” OR "babies” OR "newborn” OR "infant” OR "pediatr" OR "girl” OR (“boy” NOT “boyd”) OR “toddler” OR “pedsql” OR “ages 9” OR “4th-grade” OR “pre-school” OR “preschool”

Category	Subcategory	Phrases searched in the title or keywords[a]
		OR “pre school” OR (“primary school” NOT “teacher”) OR (“primary-school” NOT “teacher”) OR (“elementary school” NOT “teacher”) OR (“elementary-school” NOT “teacher”)
	Older people	(“aged” NOT (“school-aged” OR “schoolaged” OR “advantaged”)) OR “senior“ OR “elderl” OR “older adult” OR “older-adult” OR “older-people” OR “older people” OR “older age” OR “late life” OR “late-life” OR “older” OR “geriat” OR “old age” OR “aging adult" OR “later-life” OR “late life” OR “frail”
	Men	("^men" NOT “^mental”) or (“in men” NOT “in mental”) OR "middle-aged men" OR “(^man$” NOT “^manag”) OR "^male” OR "mascul” OR "mens health” OR "healthy-men” OR ("boy” NOT “boyd”) OR "gentleman” OR "gentlemen” OR “scottish man”
	Women	"women” OR "woman” OR "female” OR "girl” OR "lady” OR "ladies” OR "femin"
Setting	School or university	(“school” NOT “teacher”) OR (“university” NOT “teacher”) OR “college” NOT “teacher”) OR “classmate” OR “classroom” OR “curriculum” “
	Workplace	“workplace” OR “workplace” OR “work-place” OR “work-related” OR “work related” OR “work site” OR “worksite” OR “work-site” OR “worker” OR “working populat” OR “working people” OR “employer” OR “employee” OR “workstation” OR “work station” OR “office” OR “desk work” OR (“sick” AND “leave”) OR (“sick” AND “absen”) OR (“burn” AND “out”) OR “at work” OR “colleague” OR “working hour”
	Community	“communit”
	Low- or middle-income countries, or low-income settings	“developing countr” OR “low-income countr” OR “low income countr” OR “middle-income countr” OR “middle income countr” OR “LMIC” OR “low income” OR “low-income” OR

Category	Subcategory	Phrases searched in the title or keywords[a]
		“poverty” OR “poor people” OR “poor men” OR “poor women” OR “poor background” OR (any named LMIC as per Worldbank Classification)
	Family	(“family” NOT “medicin” OR “history”) OR “families”
Research methodology/focus	Trial	NOT (“review” OR “meta-analysis OR “metaanalysis”) AND ((''trial” NOT (“endometrial” OR “industrial” OR “terrestrial”)) OR “rct”[b] OR “experiment” OR intervention stud” OR “intervention proto” OR "intervention mapping proto” OR "intervention adhere" OR “complex intervention” OR (“intervention” AND “testing”) OR “randomi” OR “randomly” OR “run-in” OR “run in” OR “blind” OR “consort statement” OR “placebo” OR “drop-out” OR “dropout” OR “factorial-design” OR “factorial design”) OR “controlled study” OR “pre-post test”
	Meta-analysis or review	"metaanal” OR "meta anal” OR "meta-anal” OR ("review" NOT "reviewer") OR (“content analysis” AND (“apps” OR “applications” OR “games” OR “monitors”)
	Qualitative study	NOT (“review” OR “meta-analysis OR “metaanalysis”) AND (“qualitative” OR “focus group” OR “focus-group” OR (“motivation” AND “interview”) OR “thematic anal” OR “themes” OR “discours” OR “discursiv” OR “barrier” OR “faciltator“ OR (“belief” NOT (“model” OR “theory”)) OR (“believ” NOT (“model” OR “theory)) OR “opinion” OR “accept” OR (“experien” NOT (“flow state” OR “model” OR “theory”)) OR (“expectation” AND “changes”) OR “attitude” OR ("sentiment" NOT “sentimental") OR “usabil” OR “user cent” OR “user-cent” OR “usercent" OR “action research”) OR “narrat”
	Observational study	NOT (“review” OR “meta-analysis OR “metaanalysis”) AND ("observational stud” OR "cohort” OR "follow-up” OR

Category	Subcategory	Phrases searched in the title or keywords[a]
		"followup" OR "follow up" OR "longitudinal" OR "survey" OR "case-control" OR "case control" OR "casecontrol" OR "ecological stud" OR "cross sectional" OR "crosssectional" OR "cross-sectional" OR "survival analysis" OR "survival-analysis" OR "epidemiol" OR "10-year risk" OR "5-year survivors")
	Costs (including cost-effectiveness and financial incentives)	NOT ("review" OR "meta-analysis OR "metaanalysis") AND ("cost" OR ("economic" NOT "socio-economic" NOT "socio economic" NOT "socio economic") OR "business admin" OR "financ" OR "incentive")
	Creative methods/design	NOT ("review" OR "meta-analysis OR "metaanalysis") AND ("art-based" OR "art based" OR "artbased" OR "arts" OR ("design" AND "cent") OR "design thinking" OR "cocreat" OR "co-creat" OR "co creat" OR "participatory" OR "citizen science" OR "concept map")
	Mixed methods (including Delphi studies)	NOT ("review" OR "meta-analysis OR "metaanalysis") AND ("mixed method" OR "mixed-method" OR ("quanti" AND "qualitat") or "mixed approach" OR "multimethod" OR "multi-method" OR "multi method" OR "mixed design" OR "delphi")

[a] We conducted the search using the statistical software program Stata 14.0. The method used resulted in a hit if any part of the title or the author- and WoS-defined keywords contained any of the strings defined above. An example of a Stata code used is the following:

/ games, gamification AND augmented/virtual reality*/*

gen TI2=upper(TI)

gen key_game=0

foreach x of varlist keyword {

```
replace key_game=1 if regexm(`x', "GAME")==1& key_game~=1
replace key_game=1 if regexm(`x', "GAMING")==1& key_game~=1
replace key_game=1 if regexm(`x', "GAMIF")==1& key_game~=1
replace key_game=1 if regexm(`x', "AUGMENT")==1& regexm(`x', "REALIT")==1&key_game~=1
replace key_game=1 if regexm(`x', "VIRTUAL")==1& key_game~=1
replace key_game=1 if regexm(`x', "NINTENDO")==1& key_game~=1
replace key_game=1 if regexm(`x', "KINECT")==1& key_game~=1
replace key_game=1 if regexm(`x', "WII")==1& key_game~=1
replace key_game=1 if regexm(`x', "AVATAR")==1 & key_game~=1
replace key_game=1 if regexm(`x', "EXERGAM")==1 & key_game~=1
replace key_game=1 if regexm(`x', "ADVERGAM")==1 & key_game~=1
replace key_game=1 if regexm(`x', "POKEMON GO")==1 & key_game~=1
replace key_game=1 if regexm(`x', "ANIMAT")==1 & key_game~=1
replace key_game=1 if regexm(`x', "INTERACTIVE DANCE")==1 & key_game~=1
}
```

[b] If it equals the exact phrase only (i.e. “dance”, “fit”, “app”, “pda” or “rct” by itself)

[c] If occurring at the very end of a word only

[d] Not presented in Table 3, but included because the subcategory was used to define generation 1 technologies
