# Supplementary material for: Physical Activity, Sedentary Behavior, and Diet-Related eHealth and mHealth Research: Bibliometric Analysis
Source: J Med Internet Res. 2018 Apr 18;20(4):e122. doi: 10.2196/jmir.8954 (PMC5932335; doi:10.2196/jmir.8954)
Supplement: Multimedia Appendix 6 [file jmir_v20i4e122_app6.pdf]

Multimedia Appendix 6. Number of papers per WoS subject category and publication year included in the bibliometric analysis

WoS subject category	Publication year																	
	2000	2001	2002	2003	2004	2005	2006	2007	2008	2009	2010	2011	2012	2013	2014	2015	2016	All
Anthropology	0	0	0	0	0	0	0	0	0	0	0	0	0	0	0	1	1	2
Automation & Control Systems	0	0	0	0	0	0	0	0	0	0	0	0	0	1	0	0	0	1
Behavioral Sciences	1	0	0	0	0	0	0	0	0	0	0	0	3	0	3	1	3	11
Biochemistry & Molecular Biology	0	0	0	0	0	0	0	0	0	0	0	0	0	0	0	0	1	1
Biology	0	0	0	0	0	0	0	0	0	0	0	1	0	0	0	0	0	1
Biophysics	0	0	0	0	0	0	0	0	0	0	0	0	0	0	1	0	0	1
Biotechnology & Applied Microbiology	0	0	0	0	0	0	0	0	0	0	1	0	0	0	0	1	0	2
Business	0	0	0	0	0	0	0	0	0	0	0	1	0	2	1	0	0	4
Cardiac & Cardiovascular Systems	0	0	0	0	0	0	2	0	0	2	0	2	7	2	3	8	14	40
Cell Biology	0	0	0	0	0	0	0	0	0	0	0	1	0	0	0	0	0	1
Chemistry	0	0	0	0	0	0	0	0	0	0	0	0	0	1	1	0	4	6
Clinical Neurology	0	0	0	0	1	0	0	0	2	0	1	1	2	0	1	0	4	12
Communication	0	0	0	0	0	0	0	0	1	4	1	3	2	3	4	5	8	31
Computer Science	0	0	0	0	0	3	2	3	1	2	4	3	5	8	21	9	12	73
Critical Care Medicine	0	0	0	0	0	0	0	0	0	0	0	0	0	0	0	3	0	3
Cultural Studies	0	0	0	0	0	0	0	0	0	0	0	0	0	0	0	0	2	2
Ecology	0	0	0	0	0	0	0	1	0	0	0	0	0	0	0	0	0	1
Economics	0	0	0	0	0	0	0	0	0	0	0	0	0	0	1	1	0	2
Education & education research	3	2	2	1	2	3	7	2	10	5	6	7	7	10	11	10	15	103
Electrochemistry	0	0	0	0	0	0	0	0	0	0	0	0	0	0	1	0	2	3
Endocrinology & Metabolism	0	1	1	2	4	3	5	4	4	4	6	5	5	10	13	7	22	96
Engineering	0	0	0	0	0	0	2	0	2	0	1	1	3	1	6	3	1	20
Environmental Sciences	0	0	0	0	0	0	0	1	0	1	0	1	0	0	2	2	2	9
Ergonomics	0	0	0	0	0	0	0	0	0	0	0	0	0	0	2	0	2	4
Ethnic Studies	0	0	0	0	0	0	0	0	0	0	0	0	0	0	0	0	1	1
Family Studies	0	2	1	0	0	1	0	0	0	0	0	1	0	0	0	0	0	5
Film, Radio, Television	0	0	0	0	0	0	0	0	0	0	0	0	1	0	0	0	0	1
Food Science & Technology	0	0	0	0	0	0	0	0	0	0	0	2	0	0	0	1	1	4
Genetics & Heredity	0	0	0	0	0	0	0	0	0	0	0	0	1	0	0	1	1	3

WoS subject category	Publication year																
	2000	2001	2002	2003	2004	2005	2006	2007	2008	2009	2010	2011	2012	2013	2014	2015	2016	All
Geriatrics & Gerontology	0	0	0	0	0	0	0	0	0	0	1	2	0	2	4	2	1	12
Gerontology	0	0	1	0	0	0	0	0	0	1	1	0	1	1	4	2	0	11
Health Care Sciences & Services	0	1	0	0	5	7	3	9	10	14	19	17	31	39	43	59	89	346
Health Policy & Services	0	0	0	0	2	1	2	1	0	2	1	1	13	12	14	13	16	78
Hematology	0	0	0	0	0	0	0	0	1	0	0	0	0	0	0	0	1	2
Hospitality, Leisure, Sport & Tourism	0	0	0	1	0	0	1	0	0	1	2	1	3	3	1	4	5	22
Information Science & Library Science	0	0	0	0	0	0	0	0	1	2	2	2	1	3	5	4	4	24
Instruments & Instrumentation	0	0	0	0	0	0	0	0	0	0	0	0	0	0	1	1	2	4
Management	0	0	0	0	0	0	0	0	0	0	0	0	0	0	1	0	0	1
Mathematical & Computational Biology	0	0	0	0	0	0	0	0	0	0	0	0	1	0	2	0	2	5
Mathematics	0	0	0	0	0	0	0	0	0	0	0	1	0	0	0	0	0	1
Medical Informatics	0	1	0	0	4	6	2	7	7	11	11	13	25	29	27	23	48	214
Medicine	1	0	3	5	3	2	3	8	11	8	13	4	16	15	13	18	22	145
Multidisciplinary Sciences	0	0	0	0	0	0	0	0	0	1	0	1	0	3	4	10	9	28
Neurosciences	0	0	0	0	1	0	0	0	1	0	0	1	0	0	2	1	4	10
Nursing	0	0	0	1	1	1	2	0	1	8	3	3	3	6	11	9	10	59
Nutrition & Dietetics	7	3	2	4	6	11	12	12	10	11	15	23	18	16	39	24	34	247
Obstetrics & Gynecology	0	0	0	0	0	0	0	0	0	1	1	0	0	0	2	2	3	9
Oncology	0	0	0	0	0	0	0	0	0	1	0	1	0	3	2	7	10	24
Operations Research & Management Science	0	0	0	0	0	0	0	0	0	0	0	0	0	1	0	0	0	1
Orthopedics	0	0	0	0	0	0	0	0	0	0	1	0	0	0	1	2	6	10
Pediatrics	0	2	0	1	1	0	3	1	3	4	8	5	7	6	9	4	9	63
Peripheral Vascular Disease	0	0	0	0	0	0	0	0	0	0	0	0	0	1	0	0	1	2
Pharmacology & Pharmacy	0	0	0	0	1	0	1	2	3	0	1	1	1	1	1	1	3	16
Physics	0	0	0	0	0	0	0	0	0	0	0	1	0	0	0	0	0	1
Physiology	0	1	2	0	0	0	1	4	1	5	4	3	4	4	9	4	10	52
Political Science	0	0	0	0	0	0	0	0	1	0	0	0	0	0	0	0	0	1
Primary Health Care	0	0	0	0	0	0	0	0	0	0	0	0	0	2	2	0	0	4
Psychiatry	0	0	0	0	0	1	0	0	0	0	4	2	1	0	4	1	6	19
Psychology	0	4	1	7	1	4	6	12	7	10	22	15	22	18	20	21	47	217
Public Administration	0	0	0	0	0	0	0	0	0	0	0	0	0	0	0	1	0	1
Public, Environmental & Occupational Health	2	5	5	5	5	10	9	12	26	26	37	34	41	47	70	57	66	457
Rehabilitation	0	0	0	0	0	4	0	0	1	2	1	1	16	10	18	17	24	94
Respiratory System	0	0	0	0	0	0	1	0	1	1	0	0	1	0	3	3	3	13

WoS subject category	Publication year																	
	**2000**	**2001**	**2002**	**2003**	**2004**	**2005**	**2006**	**2007**	**2008**	**2009**	**2010**	**2011**	**2012**	**2013**	**2014**	**2015**	**2016**	**All**
Rheumatology	0	0	0	0	0	0	1	1	0	0	2	0	0	1	1	2	2	10
Robotics	0	0	0	0	0	0	0	0	0	0	0	0	0	0	0	1	0	1
Social Issues	0	0	0	0	0	0	0	0	0	0	0	0	1	0	0	0	0	1
Social Sciences	0	0	0	1	0	2	2	2	4	3	5	5	0	1	2	5	7	39
Social Work	0	0	0	0	0	0	0	0	0	0	0	0	1	0	0	0	0	1
Sociology	0	0	0	0	0	1	0	0	0	0	0	0	0	1	0	0	0	2
Sport Sciences	0	2	2	1	0	2	3	3	2	4	7	6	7	5	14	6	14	78
Substance Abuse	0	0	0	0	0	0	0	0	0	0	0	1	0	0	1	0	1	3
Surgery	0	0	0	0	0	0	0	0	0	0	0	0	0	0	0	1	0	1
Telecommunications	0	0	0	0	0	0	0	1	0	0	1	0	2	3	2	1	1	11
Toxicology	0	0	0	0	0	0	0	0	1	1	0	0	0	0	0	0	0	2
Transplantation	0	0	0	0	0	0	0	0	0	0	0	0	0	0	0	1	1	2
Transportation	0	0	0	0	0	0	1	0	0	0	0	0	1	0	0	0	0	2
Transportation Science & Technology	0	0	0	0	0	0	1	0	0	0	0	0	1	0	0	0	0	2
Urology & Nephrology	0	0	0	0	0	1	0	0	1	0	0	1	0	0	1	0	0	4
Women's studies	0	0	0	0	0	0	0	0	0	1	1	0	0	0	0	0	0	2
Total	14	24	20	29	37	63	72	86	113	136	183	174	254	271	404	360	557	2797
