# Supplementary material for: Physical Activity, Sedentary Behavior, and Diet-Related eHealth and mHealth Research: Bibliometric Analysis
Source: J Med Internet Res. 2018 Apr 18;20(4):e122. doi: 10.2196/jmir.8954 (PMC5932335; doi:10.2196/jmir.8954)
Supplement: Multimedia Appendix 7 [file jmir_v20i4e122_app7.pdf]

Multimedia Appendix 7. Publication output of countries

Country	Number of papers	Percentage of total papers
USA	836	48.83%
Australia	195	11.39%
The Netherlands	125	7.30%
England	95	5.55%
Canada	63	3.68%
Belgium	32	1.87%
South Korea	29	1.69%
Ireland	27	1.58%
New Zealand	25	1.46%
Germany	22	1.29%
Sweden	22	1.29%
China	21	1.23%
Spain	20	1.17%
Japan	19	1.11%
Finland	18	1.05%
Taiwan	17	0.99%
Italy	15	0.88%
Greece	14	0.82%
Denmark	11	0.64%
Norway	11	0.64%
Scotland	10	0.58%
Switzerland	10	0.58%
Singapore	10	0.58%
France	10	0.58%
Malaysia	7	0.41%
Saudi Arabia	4	0.23%
India	4	0.23%
Iran	4	0.23%
Israel	3	0.18%
Turkey	3	0.18%
Qatar	3	0.18%
Brazil	3	0.18%
Austria	3	0.18%
Poland	3	0.18%
Portugal	3	0.18%
Wales	2	0.12%
Thailand	2	0.12%
Lebanon	2	0.12%
Romania	2	0.12%
United Arab Emirates	1	0.06%
Nigeria	1	0.06%
Egypt	1	0.06%
Iceland	1	0.06%

Philippines	1	0.06%
Pakistan	1	0.06%
Mexico	1	0.06%
**Grand Total**	**1712**	**100.00%**
