# Supplementary material for: Physical Activity, Sedentary Behavior, and Diet-Related eHealth and mHealth Research: Bibliometric Analysis
Source: J Med Internet Res. 2018 Apr 18;20(4):e122. doi: 10.2196/jmir.8954 (PMC5932335; doi:10.2196/jmir.8954)
Supplement: Multimedia Appendix 8 [file jmir_v20i4e122_app8.pdf]

Multimedia Appendix 8. Highly-cited papers

Rank	Citation Rate[a]	Title	Design	Theme
1	79.5	Webb, T., Joseph, J., Yardley, L., & Michie, S. (2010). Using the internet to promote health behavior change: a systematic review and meta-analysis of the impact of theoretical basis, use of behavior change techniques, and mode of delivery on efficacy. *Journal of Medical Internet Research*, *12*(1), e4.	Review: systematic review & meta-analysis	Internet/Computer to improve health behaviors[b] (Internet)
2	59.6	Fjeldsoe, B. S., Marshall, A. L., & Miller, Y. D. (2009). Behavior change interventions delivered by mobile telephone short-message service. *American Journal of Preventive Medicine*, *36*(2), 165-173.	Review: systematic review	SMS/Mobile Phone/PDA to improve health behaviors[b] (SMS)
3	58.8	Cole-Lewis, H., & Kershaw, T. (2010). Text messaging as a tool for behavior change in disease prevention and management. *Epidemiologic Reviews*, *32*(1), 56-69.	Review: systematic review	SMS/Mobile Phone/PDA to improve health behaviors[b] (SMS)
4	51	Chow, C. K., Redfern, J., Hillis, G. S., Thakkar, J., Santo, K., Hackett, M. L., ... & Bompoint, S. (2015). Effect of lifestyle-focused text messaging on risk factor modification in patients with coronary heart disease: a randomized clinical trial. *JAMA*, *314*(12), 1255-1263.	Quantitative study: experimental trial	SMS/Mobile Phone/PDA to improve health behaviors[b] (SMS)
5	41	Carter, M. C., Burley, V. J., Nykjaer, C., & Cade, J. E. (2013). Adherence to a smartphone application for weight loss compared to website and paper diary: pilot randomized controlled trial. *Journal of Medical Internet Research*, *15*(4), e32.	Quantitative study: experimental trial	Various e- & mHealth to improve health behaviors[b] (app vs Internet)
5	41	Maher, C. A., Lewis, L. K., Ferrar, K., Marshall, S., De Bourdeaudhuij, I., & Vandelanotte, C. (2014). Are health behavior change interventions that use online social networks effective? A systematic review. *Journal of Medical Internet Research*, *16*(2), e40.	Review: systematic review	Social media/app/tracker to improve health behaviors[b] (social media)
7	37.2	Krebs, P., Prochaska, J. O., & Rossi, J. S. (2010). A meta-analysis of computer-tailored interventions for health behavior change. *Preventive Medicine, 51*(3), 214-221.	Review: systematic review & meta-analysis	Internet/Computer to improve health behaviors[b] (Computer)

8	37	Yardley, L., Morrison, L., Bradbury, K., & Muller, I. (2015). The person-based approach to intervention development: application to digital health-related behavior change interventions. *Journal of Medical Internet Research, 17*(1), e30.	Research methodology	Methodology for developing digital behavior change interventions
9	36	Lee, J. M., Kim, Y., & Welk, G. J. (2014). Validity of consumer-based physical activity monitors. *Medicine and Science in Sports and Exercise, 46*(9), 1840-8.	Quantitative study: validation of consumer devices	Validation of consumer-based activity trackers
10	31	Patrick, K., Raab, F., Adams, M., Dillon, L., Zabinski, M., Rock, C., ... & Norman, G. (2009). A text message-based intervention for weight loss: randomized controlled trial. *Journal of Medical Internet Research, 11*(1), e1.	Quantitative study: experimental trial	SMS/Mobile Phone/PDA to improve health behaviors[b](SMS)
11	30.8	Brouwer, W., Kroeze, W., Crutzen, R., de Nooijer, J., de Vries, N. K., Brug, J., & Oenema, A. (2011). Which intervention characteristics are related to more exposure to internet-delivered healthy lifestyle promotion interventions? A systematic review. *Journal of Medical Internet Research*, *13*(1), e2.	Review: systematic review	Internet/Computer to improve health behaviors[b] (Internet)
12	30.6	Baranowski, T., Buday, R., Thompson, D. I., & Baranowski, J. (2008). Playing for real: video games and stories for health-related behavior change. *American Journal of Preventive Medicine, 34*(1), 74-82.	Review: systematic review	Exergames/ video games to improve health behaviors[b]
13	30.5	Bort-Roig, J., Gilson, N. D., Puig-Ribera, A., Contreras, R. S., & Trost, S. G. (2014). Measuring and influencing physical activity with smartphone technology: a systematic review. *Sports Medicine, 44(*5), 671-686.	Review: systematic review	Social media/app/tracker to improve health behaviors[b] (app)
13	30.5	Kroeze, W., Werkman, A., & Brug, J. (2006). A systematic review of randomized trials on the effectiveness of computer-tailored education on physical activity and dietary behaviors. *Annals of Behavioral Medicine, 31*(3), 205-223.	Review: systematic review	Internet/Computer to improve health behaviors[b] (Computer)
15	29.2	Biddiss, E., & Irwin, J. (2010). Active video games to promote physical activity in children and youth: a systematic review. *Archives of Pediatrics & Adolescent Medicine, 164*(7), 664-672.	Review: systematic review	Exergames/ video games to improve health behaviors[b]

16	25.7	Norman, G. J., Zabinski, M. F., Adams, M. A., Rosenberg, D. E., Yaroch, A. L., & Atienza, A. A. (2007). A review of eHealth interventions for physical activity and dietary behavior change. *American Journal of Preventive Medicine, 33*(4), 336-345.	Review: systematic review	Various e- & mHealth to improve health behaviors[b] (app vs Internet)
17	24.5	Middelweerd, A., Mollee, J. S., van der Wal, C. N., Brug, J., & te Velde, S. J. (2014). Apps to promote physical activity among adults: a review and content analysis. *International Journal of Behavioral Nutrition and Physical Activity, 11*(1), 97.	Content analysis of apps	Social media/app/tracker to improve health behaviors[b] (app)
18	24	Cadmus-Bertram, L. A., Marcus, B. H., Patterson, R. E., Parker, B. A., & Morey, B. L. (2015). Randomized trial of a Fitbit-based physical activity intervention for women. *American Journal of Preventive Medicine, 49*(3), 414-418.	Quantitative study: experimental trial	Social media/app/tracker to improve health behaviors[b] (tracker)
19	23.3	Pagoto, S., Schneider, K., Jojic, M., DeBiasse, M., & Mann, D. (2013). Evidence-based strategies in weight-loss mobile apps. *American Journal of Preventive Medicine, 45*(5), 576-582.	Content analysis of apps	Social media/app/tracker to improve health behaviors[b] (app)
19	23.3	Peng, W., Crouse, J. C., & Lin, J. H. (2013). Using active video games for physical activity promotion: a systematic review of the current state of research. *Health Education & Behavior*, *40*(2), 171-192.	Review: systematic review	Exergames/ video games to improve health behaviors[b]
19	23.3	Davies, C. A., Spence, J. C., Vandelanotte, C., Caperchione, C. M., & Mummery, W. K. (2012). Meta-analysis of internet-delivered interventions to increase physical activity levels. *International Journal of Behavioral Nutrition and Physical Activity*, *9*(1), 52.	Review: systematic review & meta-analysis	Internet/Computer to improve health behaviors[b] (Internet)
22	23.1	Vandelanotte, C., Spathonis, K. M., Eakin, E. G., & Owen, N. (2007). Website-delivered physical activity interventions: A review of the literature. *American Journal of Preventive Medicine*, *33*(1), 54-64.	Review: systematic review	Internet/Computer to improve health behaviors[b] (Internet)
23	22	Cavallo, D. N., Tate, D. F., Ries, A. V., Brown, J. D., DeVellis, R. F., & Ammerman, A. S. (2012). A social media–based physical activity intervention: a randomized controlled trial. *American Journal of Preventive*	Quantitative study: experimental trial	Social media/app/tracker to improve health behaviors[b]

		*Medicine, 43(*5), 527-532.		(social media)
23	22	Conroy, D. E., Yang, C. H., & Maher, J. P. (2014). Behavior change techniques in top-ranked mobile apps for physical activity. *American Journal of Preventive Medicine*, *46*(6), 649-652.	Content analysis of apps	Social media/app/tracker to improve health behaviors[b] (app)
25	21	Azar, K. M., Lesser, L. I., Laing, B. Y., Stephens, J., Aurora, M. S., Burke, L. E., & Palaniappan, L. P. (2013). Mobile applications for weight management: theory-based content analysis. *American Journal of Preventive Medicine, 45*(5), 583-589.	Content analysis of apps	Social media/app/tracker to improve health behaviors[b] (app)
25	21	Direito, A., Dale, L. P., Shields, E., Dobson, R., Whittaker, R., & Maddison, R. (2014). Do physical activity and dietary smartphone applications incorporate evidence-based behavior change techniques? *BMC Public Health, 14*(1), 646.	Content analysis of apps	Social media/app/tracker to improve health behaviors[b] (app)
25	21	Goode, A. D., Reeves, M. M., & Eakin, E. G. (2012). Telephone-delivered interventions for physical activity and dietary behavior change: an updated systematic review. *American Journal of Preventive Medicine, 42*(1), 81-88.	Review: systematic review	SMS/Mobile Phone/PDA to improve health behaviors[b](SMS)
28	20	Turner-McGrievy, G. M., Beets, M. W., Moore, J. B., Kaczynski, A. T., Barr-Anderson, D. J., & Tate, D. F. (2013). Comparison of traditional versus mobile app self-monitoring of physical activity and dietary intake among overweight adults participating in an mHealth weight loss program. *Journal of the American Medical Informatics Association, 20*(3), 513-518.	Quantitative study: experimental trial	Social media/app/tracker to improve health behaviors[b] (app)
29	19	DeSmet, A., Van Ryckeghem, D., Compernolle, S., Baranowski, T., Thompson, D., Crombez, G., ... & Vandebosch, H. (2014). A meta-analysis of serious digital games for healthy lifestyle promotion. *Preventive Medicine, 69*, 95-107.	Review: systematic review & meta-analysis	Exergames/ video games to improve health behaviors[b]
30	18.8	Burke, L. E., Styn, M. A., Sereika, S. M., Conroy, M. B., Ye, L., Glanz, K., ... & Ewing, L. J. (2012). Using mHealth technology to enhance self-monitoring for weight loss: a randomized trial. *American Journal of Preventive Medicine*, *43*(1), 20-26.	Quantitative study: experimental trial	SMS/Mobile Phone/PDA to improve health behaviors[b] (PDA)
31	18.2	Breton, E. R., Fuemmeler, B. F., &	Content	Social

		Abroms, L. C. (2011). Weight loss—there is an app for that! But does it adhere to evidence-informed practices? *Translational Behavioral Medicine*, *1*(4), 523-529.	analysis of apps	media/app/tracker to improve health behaviors[b](app)
32	18	Eakin, E. G., Lawler, S. P., Vandelanotte, C., & Owen, N. (2007). Telephone interventions for physical activity and dietary behavior change: a systematic review. *American Journal of Preventive Medicine*, *32*(5), 419-434.	Review: systematic review	SMS/Mobile Phone/PDA to improve health behaviors[b] (PDA)
32	18	Rosenberger, M. E., Buman, M. P., Haskell, W. L., McConnell, M., & Carstensen, L. L. (2016). Twenty-four Hours of Sleep, Sedentary Behavior, and Physical Activity with Nine Wearable Devices. *Medicine and Science in Sports and Exercise, 48*(3), 457-465.	Quantitative study: validation of consumer devices	Validation of consumer-based activity trackers
34	17.7	Cowan, L. T., Van Wagenen, S. A., Brown, B. A., Hedin, R. J., Seino-Stephan, Y., Hall, P. C., & West, J. H. (2013). Apps of steel: are exercise apps providing consumers with realistic expectations? A content analysis of exercise apps for presence of behavior change theory. *Health Education & Behavior, 40*(2), 133-139.	Content analysis of apps	Social media/app/tracker to improve health behaviors[b] (app)
35	17.3	King, A. C., Hekler, E. B., Grieco, L. A., Winter, S. J., Sheats, J. L., Buman, M. P., ... & Cirimele, J. (2013). Harnessing different motivational frames via mobile phones to promote daily physical activity and reduce sedentary behavior in aging adults. *PloS One, 8*(4), e62613.	Quantitative study: experimental trial	Social media/app/tracker to improve health behaviors[b] (app)
36	15.6	Haapala, I., Barengo, N. C., Biggs, S., Surakka, L., & Manninen, P. (2009). Weight loss by mobile phone: a 1-year effectiveness study. *Public Health Nutrition*, *12*(12), 2382-2391.	Quantitative study: experimental trial	SMS/Mobile Phone/PDA to improve health behaviors[b] (SMS)
37	14	Baranowski, T., Baranowski, J., Thompson, D., Buday, R., Jago, R., Griffith, M. J., ... & Watson, K. B. (2011). Video game play, child diet, and physical activity behavior change: A randomized clinical trial. *American Journal of Preventive Medicine*, *40*(1), 33-38.	Quantitative study: experimental trial	Exergames/video games to improve health behaviors[b]
38	13	Chao, Y. Y., Scherer, Y. K., & Montgomery, C. A. (2015). Effects of	Review: systematic	Exergames/video

		using Nintendo Wii (TM) exergames in older adults: a review of the literature. *Journal of Aging and Health*, *27*(3), 379-402.	review	games to improve health behaviors[b]
39	11.8	Crutzen, R., de Nooijer, J., Brouwer, W., Oenema, A., Brug, J., & de Vries, N. K. (2011). Strategies to facilitate exposure to internet-delivered health behavior change interventions aimed at adolescents or young adults: a systematic review. *Health Education & Behavior*, *38*(1), 49-62.	Review: systematic review	Internet/Computer to improve health behaviors[b] (Internet)
40	10	Vandelanotte, C., Müller, A. M., Short, C. E., Hingle, M., Nathan, N., Williams, S. L., ... & Maher, C. A. (2016). Past, present, and future of eHealth and mHealth research to improve physical activity and dietary behaviors. *Journal of Nutrition Education and Behavior, 48*(3), 219-228.	Review: narrative review	Various e- & mHealth to improve health behaviors[b]
41	9	Livingstone, K. M., Celis-Morales, C., Navas-Carretero, S., San-Cristobal, R., O'Donovan, C. B., Forster, H., ... & Kolossa, S. (2016). Profile of European adults interested in internet-based personalised nutrition: the Food4Me study. European *Journal of Nutrition, 55*(2), 759-769.	Quantitative study: cross-sectional	Internet/Computer to improve health behaviors[b] (Internet)
42	6	Bardus, M., van Beurden, S. B., Smith, J. R., & Abraham, C. (2016). A review and content analysis of engagement, functionality, aesthetics, information quality, and change techniques in the most popular commercial apps for weight management. *International Journal of Behavioral Nutrition and Physical Activity*, *13*(1), 35.	Content analysis of apps	Social media/app/tracker to improve health behaviors[b](app)

[a] The mean number of citations per year

[b] Health behaviors included physical activity, sedentary behavior and/or diet
